# Genetic polymorphism of *myostatin* gene in Sumba Ongole (*Bos indicus*) cattle and its association with growth traits

**DOI:** 10.5455/javar.2022.i625

**Published:** 2022-11-18

**Authors:** Cynthia Dewi Gaina, Filphin Adolfin Amalo

**Affiliations:** 1 Laboratory of Veterinary Clinic, Reproduction, Pathology, and Nutrition, Faculty of Medicine and Veterinary Medicine, Universitas Nusa Cendana, Kupang, Indonesia; 2 Laboratory of Veterinary Anatomy, Physiology, Pharmacology and Biochemistry, Faculty of Medicine and Veterinary Medicine, Universitas Nusa Cendana, Kupang, Indonesia

**Keywords:** Myostatin gene, polymorphism, Sumba Ongole cattle, *Bos indicus*, growth traits

## Abstract

**Objective:**

As one of the most valuable genetic resources of Ongole beef cattle globally, the Sumba Ongole (SO) cattle population is being studied in this investigation of myostatin (*MSTN*) gene polymorphism and its association with growth traits.

**Materials and Methods:**

Blood samples from 161 SO cattle were collected and analyzed. Deoxyribonucleic acid (DNA) was isolated. The DNA was electrophoresed and extracted, and finally, the annealing temperature was optimized, followed by amplification and sequencing. Next, we used a Basic local alignment search tool to assess the sequencing data.

**Results:**

The analysis revealed 22 single nucleotide polymorphisms (SNPs) in the *MSTN* gene in this region that showed genetic variation. Two SNPs, c.424 G > A, and c.467 G > C, were found to be significantly associated with SO cattle phenotypes of wither height, heart girth, and hip height (*p* < 0.05) but not with body weight or body length (*p* > 0.05).

**Conclusion:**

As a result of our findings, the *MSTN* gene polymorphism and its correlation with growth traits in SO cattle may be employed as a candidate marker in SO cattle and other beef cattle breeds.

## Introduction

Skeletal muscle growth is controlled by myostatin (*MSTN*), sometimes called growth and differentiation factor 8 [1–4]. It has been mapped to bovine chromosome 2 and is composed of three exons and two introns [5,6]. The *MSTN* gene, which encodes a member of the transforming growth factor family, suppresses the accumulation of skeletal muscle [7,8]. In addition, it is essential for the pre-natal and post-natal development of muscle in animals, including myoblast proliferation, muscle precursors, and muscle cell differentiation [9–13]. Muscle hypertrophy is caused by the activation of the *MSTN* gene receptor, which also blocks the activity of Akt (protein kinase B), a key regulator of muscle protein synthesis and cell proliferation. MSTN expression occurs in bovine embryos when primary myoblasts unite to generate myofibers [1,9].

Some quantitative trait genetic investigations in animal populations focus on detecting single nucleotide polymorphisms (SNPs) and their polymorphisms [14,15]. It also includes an attempt to detect an association between these polymorphisms and their significance on specific species that contribute to livestock breeders’ economic growth [16–18]. Some other domestic farm animals have also been studied for gene polymorphism concerning muscle hypertrophy and growth traits [10,19,20]. It is especially significant in farm breeding because the introduction of current genotyping technologies has offered a new source of information for selecting farm animals [15,16,18]. Several domestic farm animals’ *MSTN* gene expression has been studied for associations with performance and functional features [11,21–23]. One of the most important factors in the cattle breeding industry is the *MSTN* gene and the beneficial traits it is associated with [11,24,25].

The role of the *MSTN* gene in economic production has been identified in various breeds of beef cattle, such as Belgian blue [26], Nellore cattle [25,27], Piemontese young bulls [23,28], Russian cattle [2], Marchigiana bulls [3], and, Chinese yellow cattle [29]. The trait can be found in Australian beef cattle [30] and in some breeds of Indonesian beef cattle, such as the Peranakan Ongole (PO), the Belgian Blue × PO hybrid, and the PO × Pegolo Bali cattle [31,32]. However, the result might probably be different from other Ongole beef cattle extensively grazed under a harsh environment in a tropical semi-arid region, such as Sumba Island [33]. The PO breed of cattle is a crossbreed of the Java and Sumba Ongole (SO) breeds [32], while the SO cattle are one of Indonesia’s native beef cattle breeds, brought originally from India by the Indonesian Government in the earliest of 18th century. The SO cattle have been extensively grazed on Sumba Island for genetic development [34]. Because of its benefits as a source of protein and its strong adaptability to harsh environmental conditions, this breed has been designated a nationally protected genetic resource [33,35].

The *MSTN* gene in beef cattle is polymorphic in previous studies [2,24,36]. Increased muscle mass and decreased fatness have both been linked to a shift in the amino acid position of leucine in the *MSTN* gene in European crossbreds such as Limousine and Charolais cattle [37], growing beef heifer [24], and Australian Limousine cattle [36]. European cattle breeds with heterozygous genotype variations, such as Limousine, exhibited better meat quality and growth performance than homozygous genotypes. However, it has been established that *MSTN* gene polymorphism and its association with growth traits in European cattle breeds [37], Angus cattle [11,12], Belgian Blue cattle [26], and Nellore cattle [25,27] and the association between the *MSTN* gene polymorphism and growth traits in SO cattle, one of the *Bos indicus* breed, have not been studied in depth. To date, the *MSTN* gene is considered polymorphic in other Ongole crossbred cattle, such as PO cattle, Bengali, or Zebu cattle (*B. indicus*) [32].

As it is known that the *MSTN* gene is related to productive qualities in other breeds of cattle, it is necessary to analyze in detail the role of the *MSTN* gene in SO cattle. The biological aspects of the gene have yet to be completely understood, despite its importance in beef cattle muscle development, carcass, and meat quality [25,26,30,38]. As one of the valuable genetic resources of Ongole beef cattle, this study seeks to evaluate the genetic variation of the *MSTN* gene and its association with growth traits in a population of SO cattle. The results are expected to be a potential source for molecular markers in other Ongole cattle. Hence, this study provides exciting new opportunities as an initial investigation of the *MSTN* gene in Ongole cattle.

## Materials and Methods

### Ethical approval

The Animal Research Ethics Committee of the Faculty of Veterinary Medicine, Universitas Nusa Cendana, approved this study (KEH/FKH/NEPH/2019/003).

### Deoxyribonucleic acid (DNA) and sample collecting

The blood of 161 free-ranged SO cattle on Sumba Island, NTT was collected. Each venoject needle was attached to a vacutainer ethylenediamine tetraacetic acid (EDTA) tube, and it was applied to collect blood samples from the jugular and coccygeal veins. About 3–5 ml of blood was collected and stored at 4°C until further study, such as DNA extraction, amplification, and data analysis, could be performed. This extracted DNA was assessed with a spectrophotometer and electrophoresed in agarose gel.

### Traits measurements

Some of the direct measurements of growth traits were based on [39,40]. This includes assessment variables like height, weight, chest circumference, hip circumference, and length. This assessment was performed using a measuring tape and a modified caliper.

### Polymerase chain reaction (PCR) primers and amplification

PCR, electrophoresis, and DNA sequencing were the methods employed. Following a 1 min pre-denaturation step at 95°C, the samples were subjected to 35 cycles of denaturation for 15 sec at 95°C, annealing for 15 sec at 58°C, and extension for 10 sec at 72°C. The final period of the extension was conducted for 1 min at 72°C. The *MSTN* gene primer was used to amplify the DNA in each sample for 35 cycles. Afterward, it was analyzed using a 1.5% agarose gel in a 0.5 Tris-borate-EDTA (TBE) buffer and a 100-bp ladder to determine its molecular weight. About 2 μl of DNA template, 10 μl of nuclease-free water, 0.5 μl of each forward and reverse primer, and 12.5 μl of MyTaq HS red Mix 2x were used in a 25 μl reaction. A piece of the *MSTN* gene was amplified using the primers. Based on the sequence of the bovine *MSTN* gene, we designed the forward primer 5’-GATTGATATGTAGGTGTTCG-3’ and the reverse primer 5’-AGGGCTACCACTGGGG-3’ (GenBank Acc. No. AY794986.1).

### Analysis of sequencing

1^st^ Malaysia sequenced PCR products encoding various genotypes for each gene. BioEdit was used to analyze the sequencing results. Meanwhile, the final step in identifying the amplified nucleotide is sequencing. Molecular evolutionary genetics analysis version 6.0 was applied to discover whether nucleotide mutations occurred [23]. Then, the software Basic local alignment search tool (BLAST) was used to scan the GenBank database from National center for biotechnology information (NCBI) for reference and homologous sequences.

### Statistical analysis

The data were analyzed using IBM SPSS Statistics for Windows, Version 20.0 (IBM Corp, Armonk, NY). To examine the *MSTN* gene variants in the SO cattle population, the genetic parameters were measured. The *MSTN* gene shows the variance in the SO cattle population as indicated by the genotype and allele frequencies. Using methods based on those developed by Nei [41], population genetic diversity indices such as heterozygosity, Hardy–Weinberg equilibrium, and the polymorphism information content (PIC) were constructed. PIC is a polymorphism indicator. Genetic variants have low, medium, and high genetic diversity based on PIC values of < 0.25, 0.5 > PIC > 0.25, and PIC > 0.5, respectively [42]. The association between MSTN and growth traits was also modeled using the general linear model.

## Results

### DNA amplification

**Figure 1.**
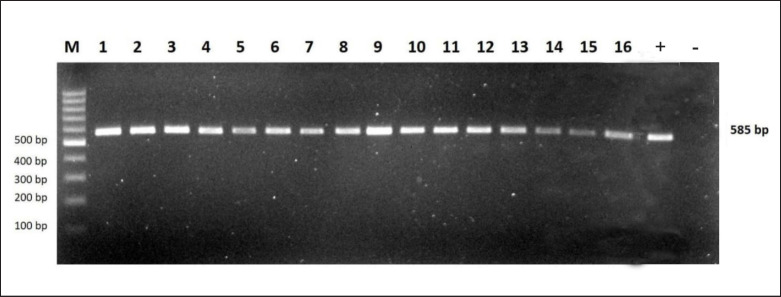
PCR amplification of *MSTN* gene of Sumba Ongole (*B. indicus*) cattle. +, positive control, −, Negative control.

The coding region of the *MSTN* gene was designed to amplify by PCR. Sequencing cannot begin until the DNA tube is cut up and amplified. The data suggested that the DNA fragment that was amplified was 585 bp in length. The *MSTN* gene PCR products were amplified in a thermocycler at a temperature of 58°C for 15 sec, and then they were identified on a 1.5% TBE agarose gel. This result confirmed the high specificity of the amplified fragment, which was then made available for sequencing. Figure 1 is a graphical representation of the PCR results.

The primers are specific to the *MSTN* gene, with just one DNA band produced in all samples (Fig. 1). The primers used in PCR were two oligonucleotides that had the same sequence as the DNA template. Furthermore, the sequencing results were evaluated on NCBI using the BLAST nucleotide software. The BLAST program’s main step is to identify and align the DNA sequences in GenBank that are most similar to the sample’s sequences.

For all samples, the annealing temperature of 58°C for 15 sec significantly affects the success of the amplification process. The annealing temperature is the temperature at which the primers will connect to the amplified target DNA with the highest efficiency during the PCR. This diversity is due to differences in PCR machine parameters and reagent composition. Primers attenuate at temperatures between 36°C and 72°C, with 50°C–60°C being more typical [43]. The concentrations of the primer and the gene being targeted, as well as the volume of the PCR reaction, determine how long the annealing time must be for the primer to bind to its complementary target [43]. Primer extension at 72°Cfor 15 sec lengthens the primer linked to the subsequent target. Final elongation lasted 2 min at a steady state. Denaturation, annealing, and elongation are the three steps of PCR that constitute 1 thermal cycle, and in this study, a total of 35 cycles were conducted. According to [44], when the length of the target molecule reaches 1.0 × 10^3^, the required cycles are 35–40.

### MSTN gene genotype distribution, allelic abundance, heterozygosity, and Hardy–Weinberg equilibrium

Sequencing analysis of the *MSTN* gene in SO cattle produced 22 polymorphic SNPs, namely, c.52 A > C, c.351 C > T, c.357 C > A, c.377 T > C, c.424 G > A, c.428 A > G, c.435 G > C, c.442 G > A, c.455 G > C, c.457 A > G, c.460 C > G, c.467 G > C, c.490 C > G, c.491 A > G, c.495 T > C, c.500 T > C, c.503 C > A, c.508 C > G, c.531 G > A, c.576 A > G, and c.583 G > A. To be considered polymorphic, a SNP must have an allele frequency of ≤ 0.99 in big populations and ≤ 0.95 in small populations [41]. A molecular selection marker in cattle is required for assessing genetic diversity. Following the criteria of [42], the PIC value 0.5 > PIC > 0.25 was reached from low (0.04) to high (0.68). A total of 3 SNPs in the *MSTN* gene with a high PIC value was c.351 C > T, c.457 A > G, and c.500 T > C as recapitulated in Table 1.

### Association MSTN gene with growth traits

Table 2 shows that the SO cattle’s wither height, heart girth, and hip height were significantly (*p* < 0.05) related to two polymorphic SNPs in the *MSTN* gene: c.424 G > A and c.467 G > C. In contrast, there was no statistically significant correlation between MSTN and body weight and body length (*p* > 0.05).

## Discussion

The association between *MSTN* gene polymorphism and growth traits in SO cattle, one of the Ongole Breeds, has never before been studied as it is stated that the polymorphisms discovered to offer the possibility of genetic variation and productive traits that might be utilized for breed differentiation [38,45]. A polymorphism is a change in the DNA sequence that occurs in 1% or more of a population [46]. Polymorphism is induced by one or more changes in the order of nucleotides in a gene [24,47]. Mutation, migration, and selection are all variables that influence nucleotide composition [38,45]. These alterations have an impact on an organism’s phenotype [48]. The most common form of mutation in the mammalian genome is known as an SNP, which is a DNA sequence polymorphism that occurs at significant levels in a population [9,20,49,50].

A SNP is termed polymorphic when the frequency of at least one of its alleles is less than 0.99 in large populations and less than 0.95 in small groups [41]. How often a certain allele occurs in a certain population is expressed as a percentage of the total number of alleles [48]. Meanwhile, the genotype frequency demonstrates the genotype frequency distribution by calculating the frequency distribution of a set of genotypes across populations [14]. Single-nucleotide polymorphisms are helpful as genetic markers in populations for studying their susceptibility to specific features, especially in animal production [41,46,51]. A polymorphism may have spontaneously developed with a neutral or advantageous impact if its prevalence is higher in the population [13,16,19,37].

Considering the importance of this gene, investigation of the biological roles of the *MSTN* gene in Ongole cattle is required because mutations in some of the other beef cattle, including European cattle [23,27,28], Australian breeds [30], and Indonesian beef cattle [31,32,35] are associated with growth traits. The literature search revealed that there is no prior research examining the allelic and genotypic frequencies of the *MSTN* gene in SO cattle, making direct comparisons with other studies in Ongole cow breeds worldwide difficult. Multiple *MSTN* gene polymorphisms were identified in SO cattle, however, according to this investigation (Table 1). The *MSTN* gene in SO cattle was sequenced, and the results revealed 22 SNPs with variable frequencies, namely c.52 A > C, c.351 C > T, c.357 C > A, c.377 T > C, c.424 G > A, c.428 A > G, c.435 G > C, c.442 G > A, c.455 G > C, c.457 A > G, c.460 C > G, c.467 G > C, c.490 C > G, c.491 A > G, c.495 T > C, c.500 T > C, c.503 C > A, c.508 C > G, c.531 G > A, c.576 A > G, and c.583 G > A. The SNPs in this study had an allele frequency of less than 0.99 or greater than 0.01. In addition, this finding investigated that the highest allele frequency namely 0.98 was found in SNP c.52 A > C, c.357 C > A, c.377 T > C, c.424 G > A, c.428 A > G, c.435 G > C, c.442 G > A, c.455 G > C, c.490 C > G, c.508 C > G, and c.583 G > A.

**Table 1.** Polymorphism of *MSTN* gene in SO cattle (*B. indicus*).

SNPs	Genotype frequencies			Allele frequencies		He	Ho	X^2^	Pic
c.351 C>T	CC	CT	TT	C	T				
	0.574	0.064	0.362	0.61	0.39	0.48	0.06	0.155	0.63
c.457 A>G	AA	AG	GG	A	G				
	0.553	0.064	0.383	0.59	0.41	0.49	0.06	0.172	0.66
c.500 T>C	TT	TC	CC	T	C				
	0.532	0.064	0.404	0.56	0.44	0.04	0.06	0.190	0.68

Note: He = expected heterozygosity; Ho = observed heterozygosis; *χ*^2^ = Hardy–Weinberg equilibrium; not significant (ns) at α 5% (χ^2^ obs ≤ 5.59); *n* = 161 heads.

**Table 2.** Association of SNPs MTSN gene with growth traits in SO cattle (*B. indicus*).

SNPs	G	*n*	Body length (cm)	Wither height (cm)	Heart girth (cm)	Hip height (cm)	Body weight (kg)
c.424 G>A	GG	45	129.91 ± 7.88	124.39 ± 5.32	153.87^a^ ± 11.12	132.85^ b^ ± 5.27	113.55 ± 2.45
	GA	2	125.5 ± 13.44	119.5 ± 3.54	144.00 ± 7.07	128.00 ± 2.83	112.00 ± 4.24
	AA	0	0.00 ± 0.00	0.00 ± 0.00	0.00 ± 0.00	0.00 ± 0.00	0.00 ± 0.00
c.467 G>C	GG	42	129.6 ± 8.01	123.93 ± 4.79	152.98 ± 10.72	132.29 ± 4.68	113.45 ± 2.48
	GC	0	0.00 ± 0.00	0.00 ± 0.00	0.00 ± 0.00	0.00 ± 0.00	0.00 ± 0.00
	CC	5	133.6 ± 5.5	127.8 ± 8.23	160.40^ b^ ± 13.54	137.00^ a^ ± 8.09	114.70 ± 1.89

Note: cells with different letters. ^a,b^ differed significantly (*p* < 0.05); G: Genotype; *n*: total numbers of SO cattle.

Based on the results, mutations of the *MSTN* gene provide potential opportunities as a molecular marker for improving productivity performance in SO cattle breeding as in other beef cattle [24]. Thus, the polymorphism in SO cattle in this study might contribute to the differences in the composition of the MSTN gene, as indicated by the size of the DNA fragment. The mutation of MSTN in other cattle breeds is related to the double muscling trait, but mutation in SO cattle in this study might contribute to the growth and hypertrophy of muscles [3,29,38]. Further investigation is required to determine whether this mutation affects the phenotype of Ongole cattle. However, the gene polymorphism might be related to the species-specific type of animals [52,53]. A previous study discovered that the *MSTN* gene mutation in Bali cattle is most likely caused by their ability to adapt to a range of harsh environmental situations [54]. Point mutations are DNA alterations that occur when a single nucleotide is inserted, deleted, or replaced [14]. As a result, the whole structure of a chromosome is changed, either by flipping, deleting, duplicating, or translocating it [55]. Hardy–Weinberg equilibrium is affected by selection, non-random mating, mutation, genetic drift, and migration [53].

It is clear from Table 1 that there was a strong selective pressure at work in this population since the observed heterozygosis (Ho) was lower than the expected heterozygosis (He). Furthermore, it demonstrated that genotype imbalance in observed SO cattle may be measured by comparing the Ho value to the He value for heterozygosis. Possible implications of this finding include the existence of a selection activity and the lack of random mating. Since an SNP is regarded to have high diversity when the heterozygosis value is > 0.50 [52,53]. The heterozygosity value in the SO cattle indicates that all SNPs are under low diversity circumstances with Ho ≤0.5. Heterozygosity levels are a useful indicator of a population’s genetic variation, which aids the selection process [56]. It is also a potential referral source for selecting breeding programs when diverse populations and crosses are well performed in a uniform population [18].

Evaluation of genetic diversity in cattle requires using a molecular selection marker [18,27,38,45]. Following the criteria of [41], the PIC value of 0.25 ≤ PIC ≤ 0.5 in this study was reached from low (0.04) to high (0.68). A total of 3 SNPs in the *MSTN* gene had high PIC values of c.351 C > T, c.457 A > G, and c.500 T > C (Table 1). PIC can be used to identify genetic markers and the presence or absence of polymorphic alleles (along with the heterozygosity value, of course). Results indicated variation in the *MSTN* gene, indicating promising utility for molecular selection in breeding. Investigation of candidate gene association is the initial step toward understanding the genetic basis of economically relevant characteristics [18,27,38,45]. An important reason for assessing the effects of SNP associations in the beef industry is that the connections within diverse interactions will culminate in significant genotypic or phenotypic alterations [18,24].

The relationship between polymorphisms and the efficiency of growth traits in SO cattle has never before been studied. Consistently, this investigation’s results pointed toward the sheep MSTN sequence being most analogous to the cattle MSTN sequence [10,57,58] and goats [7,10,21]. Researchers found no evidence that the *MSTN* gene played a role in the weight of SO cattle (*p* > 0.05) (Table 2).

Similar to other studies, this one demonstrated that the *MSTN* gene was not significantly associated with body weight (*p* > 0.05) in Friesian cattle [16], in growing beef heifers [24], and Shaanbei White Cashmere goat [10] during pre- and post-natal muscle development. Conversely, birth weight, corrected weaning weight, and different age weights are significantly impacted by *MSTN* gene mutations in European cattle breeds such as Piedmont, Angus, and Hayford [6].

One of the Chinese cow breeds, Qinchuan, had its *MSTN* gene found to be significantly (*p* < 0.05) linked to total body length [59]. However, SO cattle did not show a statistically significant difference in body length (*p >* 0.05). These results imply that the *MSTN* gene is essential for regulating muscle development but that several other genes (polygenes) are also involved. These include calpastatin, leptin, growth hormone, growth hormone receptor, pituitary transcription factor (Pit-1) [16,47], and Heat Shock Protein 70-1 gene [60] also influence growth traits which are primarily influenced by environmental factor[58].

Based on the results, two polymorphic SNPs, c.424 G > A, and c.467 G > C, were significantly (*p* < 0.05) associated with wither height, heart girth, and hip height in SO cattle, as demonstrated in Table 2. Our results were consistent with previous studies that showed the mutation of the *MSTN* gene was significantly (*p <* 0.05) related to body height, heart girth, and hip height in Shaanbei White Cashmere goat kids [10] in Chinese Dabieshan cattle [29,61], and Nanyang cattle (*B. indicus*) as one of the best Chinese cattle breeds [59]. It suggested that this mutation is a potentially useful genetic marker for other domestic farm animals. Since the *MSTN* gene’s major role is to regulate skeletal muscle development [14,27]. There is a correlation between growth features and a polymorphism that alters the amino acid sequence of MSTN in SO cattle. The findings suggest that MSTN is a key regulator of growth in Ongole beef cattle and could serve as a benchmark for other cattle breeds. This association can become a candidate marker for improving productivity in Ongole cattle.

## Conclusion

For the first time, the *MSTN* gene was isolated and characterized in SO cattle. A total of 22 polymorphic SNPs were obtained in which two namely c.424 G > A and c.467 G > C were significantly associated with wither height, heart girth, and hip height in the SO cattle population. Studying the gene related to growth traits in Ongole beef cattle is crucial to support breeding programs for improving production efficiency, reducing time and cost, and leading to optimal genetic selection. With these findings, we may take the first step in improving productivity in SO cattle by pinpointing the role played by the *MSTN* gene. That conclusion has to be confirmed by larger-scale studies since the *MSTN* gene’s whole genomic structure, which was identified by SNPs, may be more important for production in SO cattle and other Ongole beef cattle.

## List of abbreviations

BLAST, Basic local alignment search tool; DNA, Deoxyribonucleic acid; EDTA, Ethylenediamine tetra acetic acid; NCBI, National center for biotechnology information; PCR, Polymerase chain reaction; PIC, polymorphism information content.
